# Functional analysis for gut microbes of the brown tree frog (*Polypedates megacephalus*) in artificial hibernation

**DOI:** 10.1186/s12864-016-3318-6

**Published:** 2016-12-22

**Authors:** Francis Cheng-Hsuan Weng, Yi-Ju Yang, Daryi Wang

**Affiliations:** 1 Biodiversity Research Center, Academia Sinica, Taipei, 115 Taiwan; 2 Department of Life Science, National Taiwan Normal University, Taipei, 115 Taiwan; 3 Biodiversity Program, Taiwan International Graduate Program, Academia Sinica, and National Taiwan Normal University, Taipei, 115 Taiwan; 4 Department of Natural Resources and Environmental Studies, College of Environmental Studies, National Dong Hwa University, Hualien, 97401 Taiwan

**Keywords:** Artificial hibernation, Gut microbiota, Amphibian, NGS

## Abstract

**Background:**

Annual hibernation is an adaptation that helps many animals conserve energy during food shortage in winter. This natural cycle is also accompanied by a remodeling of the intestinal immune system, which is an aspect of host biology that is both influenced by, and can itself influence, the microbiota. In amphibians, the bacteria in the intestinal tract show a drop in bacterial counts. The proportion of pathogenic bacteria is greater in hibernating frogs than that found in nonhibernating frogs. This suggests that some intestinal gut microbes in amphibians can be maintained and may contribute to the functions in this closed ecosystem during hibernation. However, these results were derived from culture-based approaches that only covered a small portion of bacteria in the intestinal tract.

**Methods:**

In this study, we use a more comprehensive analysis, including bacterial appearance and functional prediction, to reveal the global changes in gut microbiota during artificial hibernation via high-throughput sequencing technology.

**Results:**

Our results suggest that artificial hibernation in the brown tree frog (*Polypedates megacephalus*) could reduce microbial diversity, and artificially hibernating frogs tend to harbor core operational taxonomic units that are rarely distributed among nonhibernating frogs. In addition, artificial hibernation increased significantly the relative abundance of the red-leg syndrome-related pathogenic genus *Citrobacter*. Furthermore, functional predictions via PICRUSt and Tax4Fun suggested that artificial hibernation has effects on metabolism, disease, signal transduction, bacterial infection, and primary immunodeficiency.

**Conclusions:**

We infer that artificial hibernation may impose potential effects on primary immunodeficiency and increase the risk of bacterial infections in the brown tree frog.

**Electronic supplementary material:**

The online version of this article (doi:10.1186/s12864-016-3318-6) contains supplementary material, which is available to authorized users.

## Background

The gut mucosal immune system forms the largest vertebrate immune compartment [1]. It is well established that the immune function depends partly on the presence of intestinal microbes. The intestinal microbiota can develop a natural defense barrier exerting different protective, structural, and metabolic effects on the host epithelium [2, 3]. The composition of the microbial community reflects the coevolution of host and microbes to achieve a balanced mutually beneficial state. Intestinal bacteria benefit from a stable environment and the host gains digestive and metabolic capabilities. It has been clearly demonstrated that diet has a considerable effect on the composition of the gut microbiota [4]. There is now mounting evidence that the microbiota is altered in people with allergies and asthma [5, 6]. Daily consumption of fermented foods may be important for maintaining the necessary amount of *Lactobacillus* bacteria and may diminish the prevalence of allergic disease. In communities in which consumption of fermented foods is high and antibiotics are not used, causes of allergy and asthma are low. These studies suggest that changes in diet and associated changes in the gut microbiota are driving the increasing incidence of inflammatory disease.

Hibernators, such as the Syrian hamster, ground squirrel, and brown bear, have been shown to restructure gut microbiota during hibernation [7–9]. Slow metabolism, nutrient turnover, and wide variation of temperature can support a dense system of anaerobic bacteria. For example, *Akkermansia muciniphila*, of the phylum Verrucomicrobia, is increased during hibernation in the Syrian hamster and ground squirrel. However, in larger animals, such as free-ranging brown bears, Verrucomicrobia, including *Akkermansia muciniphila*, are decreased during hibernation. The gut microbiota turnover of hibernators can further modulate metabolic functions. For example, the molar proportion of acetate ions rose during hibernation, which may be due to the activity of mucolytic bacteria, such as *Akkermansia muciniphila*, that convert mucins to acetate ions [10]. *Akkermansia muciniphila* has been studied to associate with the mucus layer and is able to grow on mucin as its sole carbon and nitrogen source [10–12]. Other evidence in brown bears shows that *Bacteroides fragilis* is the predominant bacterium in the microbiota from the hibernating bear, whereas both *Streptococcus* and TM7 are reduced during hibernation. Furthermore, two studies of calorie-restricted mice reported an increase in *Bacteroides fragilis* [13] but decrease in *Streptococcaceae* and TM7 [14]. Together, these data indicate that many of the changes in the brown bear microbiota are associated with calorie restriction. This suggests that the changes in gut microbiota contribute to host metabolism in the hibernators. On the other hand, colonization of the intestinal tract with diverse microbes has a profound influence on the development and function of both innate and adaptive branches of the immune system. For instance, in ground squirrels, numbers of intraepithelial lymphocytes and lamina propria leukocytes (LPL) were greater in hibernators compared with their level in summer. Compared with the summer levels, the percentage of B cells was higher and the percentage of T cells was lower in the hibernator LPL. Mucosal IgA levels were greater in entrance and torpid hibernators compared with summer levels. The results suggest that hibernation in ground squirrels is accompanied by a remodeling of the intestinal immune system, which is an aspect of host biology that is both influenced by, and can itself influence, the microbiota [15].

In amphibians, studies have shown that artificial hibernation of northern leopard frogs (*Rana pipiens*) and bullfrogs also led to a drop in bacterial counts and a change in the composition of gut microbiota [16, 17]. Hibernating northern leopard frogs [18] and chilled southern bullfrogs (*Rana catesbiana*) [17] had fewer types of facultative bacteria than that in control warm frogs. More importantly, potentially pathogenic facultative bacteria in the intestine contribute to septicemia during hibernation. For example, facultative (preferentially aerobic but facultatively anaerobic) bacteria from the intestines of frogs have been investigated as a source of septicemia, often associated with chilling and hibernation [17, 19], which occasionally kills large numbers of frogs in the laboratory and in the wild [19, 20]. Carr et al. [17] and Gibbs et al. [19] also found that hibernation can alter the relative concentrations and proportions of facultative versus anaerobic bacteria, leading to disease. Furthermore, indigenous anaerobic bacteria have been shown to control the colonization of facultative bacteria in the intestine of birds and mammals [21, 22], suggesting that species composition is positively correlated with ecosystem functioning. Therefore, characterizing microbial composition during hibernation appears to be crucial in ecological functions in amphibian guts.

Amplicon-based sequencing of marker genes is widely used for large-scale studies that involve many different sampling sites or time series. The conventional 16S rRNA gene-based analysis is a powerful tool for assessing microbial composition, but does not provide insight into the metabolic potential in the microbial communities. Therefore, the prediction of the functional capabilities of a microbial community based on marker gene data would be highly beneficial. In this study, we use high-throughput sequencing technology to provide a comprehensive analysis to characterize the shift of gut microbes before and after artificial hibernation. We first distinguish the difference of dominant gut microbes between artificially hibernating frogs (AH frogs) and nonhibernating frogs (NH frogs). In addition, the relative abundances of both potentially pathogenic facultative and anaerobic bacteria were characterized. Furthermore, we predicted the gene content of a microbial community from a marker gene survey via PICRUSt (phylogenetic investigation of communities by reconstruction of unobserved states) [23] and Tax4Fun [24] to infer a functional profile. This study demonstrates that the overall shifts in both gut microbiota and functions are affected by artificial hibernation in the brown tree frog.

## Methods

NH frogs were collected from Wazihwei Nature Reserve (121.41432° E, 25.16775° N) and private botanic gardens (120.31423° E, 23.53302° N) in the wild (snout–vent length (SVL), 2.8–8.3 cm), where the population of brown tree frogs is widely distributed in Taiwan. Individuals were collected in three distinct seasons (Table 1): 12, 18, and six individuals in fall (October and November, 2013), winter (December 2013 to February 2014), and spring (March, 2014), respectively.

**Table 1:** Summary of sample information for AH frogs and NH frogs

	NH frogs			AH frogs
	Fall	Winter	Spring	4 °C
Sample size (*N*)	12	18	6	3
Snout–vent length (cm)	5.7 ± 0.6	5.6 ± 1.3	6.5 ± 1.0	5.4 ± 0.4
Body mass (g)	12.2 ± 4.4	11.8 ± 6.3	18.7 ± 13.1	8.1 ± 2.2

*Abbreviations*: *AH frogs*artificially hibernating frogs, *NH frogs* nonhibernating frogs. Values are means ± SE

Artificial hibernation was implemented in the laboratory as used in previous studies [16, 25, 26]. After capture, we designed a lab husbandry system in the tank with the size of 90 × 45 × 60 cm^3^ and filled each tank with 5 L of water. Ivies in tanks were used as frogs’ hiding spots and facilitated the exchange of oxygen and carbon dioxide. The frogs were housed in a 23 °C room temperature with an 8:16-h light–dark cycle for at least 3 months before artificial hibernation. Frogs were fed twice a week. We used Turkestan cockroach nymphs, as they are feeder insects and a good source of protein [27]. The total feeding load is ~3% weight/body weight daily [28]. Fresh dechlorinated water was replaced every 2 days. After 3 months of acclimation, frogs were deprived of food for 1 week and all fasting frogs were then transferred into an incubator in constant darkness. The incubator always maintained a relative humidity of 90% for the AH frogs. The temperature was decreased by 5 °C every 12 h until reaching 4 °C, and remained at this value for a week.

The SVL and body weight of all individuals (both NH and AH frogs) were measured (Table 1). Fecal contents were collected from the large intestine. The anatomical site of the large intestine of the frog was clearly described in [29]. Bacterial DNA was extracted from each thawed stool sample using the QIAamp® DNA Stool Mini Kit (Qiagen, GmbH, Hilden, Germany). Before extraction, feces were loaded into a bead tube with 15 min of vibration to increase the efficiency of DNA extraction for each sample. The remaining procedures were performed according to the manufacturer’s protocol. Concentrations of double stranded DNA in the extracts were determined by the Quant-iT dsDNA HS assay kit and the Qubit fluorometer (Invitrogen, Life Technologies, Carlsbad, CA, USA). All procedures were performed in a laminar flow cabinet to avoid contamination.

For NH frogs, we used barcoding pyrosequencing to determine microbial community composition. The primer set was 515 F/806R, which targets the V4 region of the 16S ribosomal RNA gene, found to be well suited to the phylogenetic analysis of pyrosequencing reads [30]. Considering that previous studies described sources of errors in 454 sequencing runs, the valid reads should comply with appropriate rules to remove the mismatch sequences. We followed the MOTHUR [31] pipeline, which was especially designed for 454 sequencing to perform operational taxonomic unit (OTU)-based analyses. Each pyrosequencing read containing a primer sequence should be 300–350 bp in length, have no ambiguous bases, and match the 5′ primer and one of the used barcode sequences. These pyrosequencing reads were simplified using the “unique.seqs” command to generate a unique set of sequences, and then were aligned using the “align.seqs” command and compared with the Bacterial and Archaeal RDP database (RDP version 9). The aligned sequences were further trimmed and the redundant reads were eliminated using the “unique.seqs.” The remaining sequences were assigned to OTUs using the RDP classifier [32]. Only OTUs containing 0.001% of the total number of sequences were used in the analyses. The “chimera.slayer” command was used to determine chimeric sequences.

For AH frogs, we used Illumina pair-end sequencing for microbial 16S rRNA gene amplicon. The primer set was 515 F/806R, which was the same as for NH frogs. All sequences were analyzed via the Quantitative Insights Into Microbial Ecology (QIIME) pipeline [33]. Sequence reads that had ambiguous bases, had a quality score <25, had an unreadable barcode, more than one mismatch to primer sequences, did not contain the primer sequences, or <200 bp in length were removed. The remaining sequences were clustered by UCLUST [34] at a 97% similarity cutoff. The representative sequences were picked and aligned using using PyNAST [35] and taxonomy was assigned using UCLUST.

Bray–Curtis dissimilarity distances were calculated among individual samples to determine the differences in bacterial community composition across individuals [36]. Beta-diversity patterns were visualized using the NMDS (nonmetric multidimensional scaling) ordination approach [37]. A two-way nested ANOSIM (analysis of similarity) was also used to test for significant differences in bacterial composition.

To examine the relationship of gut microbiota between NH and AH frogs, Venn diagrams were created using the R package to visualize the OTUs that were shared between NH and AH frogs. Student’s *t*-test was used to compare the abundance changes between NH and AH frogs. Only differences for which *p*-value < 0.05 are reported.

To reveal the potential pathogenicity, several bacteria were selected for comparison. For example, facultative and anaerobic bacteria that were found in amphibians as shown in Banas et al. [25], in brief, anaerobic genera in the frog included *Bacteroides*, *Clostridium*, *Eubacterium*, *Fusobacterium*, *Peptococcus*, *Peptostreptococcus*, *Propionibacterium*, and *Ruminococcus*, and facultative genera included *Azotobacter*, *Bacillus*, *Corynebacterium*, *Enterococcus*, *Flavobacterium*, *Lactobacillus*, *Pseudomonas*, and *Streptococcus*. There are several pathogenic bacteria associated with red-leg syndrome (RLS)–one of the main infectious diseases that affects amphibians and causes high mortality [38]. The etiological agents involve *Aeromonas hydrophila*, *Citrobacter freundii*, *Chryseobacterium indolgenes*, *Edwardsiella tarda*, *Proteus mirabilis*, *Proteus vulgaris*, *Pseudomonas aeruginosa*, *Staphylococcus epidermidis*, and *Streptococcus iniae* [39–43].

We implemented both PICRUSt and Tax4Fun to predict the functional shifts in AH frogs. The PICRUSt approach was proposed to predict KEGG Ortholog (KO) functional profiles of microbial communities using 16S rRNA gene sequences [23]. This algorithm uses a phylogenetic tree of 16S rRNA gene sequences to link OTUs with gene content. Thus, PICRUSt predictions depend on the topology of the tree and the distance to the next organism, where a nearest neighbor within the tree topology always exists, even if distances are large. Therefore, we also apply Tax4Fun, which links 16S rRNA gene sequences with the functional annotation of sequenced prokaryotic genomes, which is realized with a nearest-neighbor identification based on a minimum 16S rRNA sequence similarity [24]. Wilcoxon’s test was used to compare the relative abundance changes between NH and AH frogs. Only differences for which *p*-value < 0.05 are reported.

## Results

### Distinct alpha diversity between AH and NH frogs

**Fig. 1:**
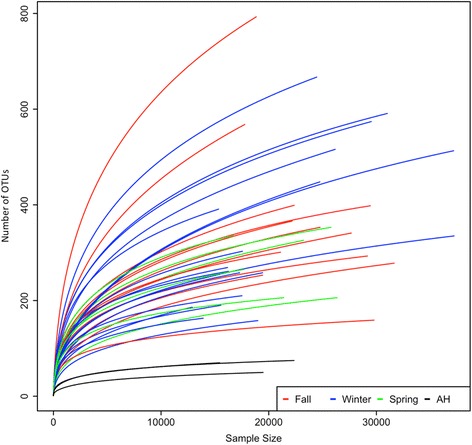
Alpha-diversity rarefaction plot of fecal microbiotas between AH frogs and NH frogs (fall, winter, and spring). The *X*- and *Y*-axes represent sample size and number of observed OTUs, respectively. *Red*, *blue*, *green*, and *black* colors refer to fall, winter, spring, and AH (4 °C), respectively. AH frogs differ significantly from NH frogs (*p*-value < 0.05)

Our sequencing reads resulted in an average of 21,289 ± 6300 high-quality sequences per sample for the colonic samples in NH and AH frogs. The observed OTUs of all AH frogs are fewer than those in NH frogs (Fig. 1). Fasting and low temperature (4 °C) are two major factors that alter microbial diversity. Here, we utilized the phylogenetic index to reveal the change in phylogenetic diversity between AH and NH frogs. Overall, the average richness was greatest in the fall, and lowest in the artificial hibernation (Table 2). All phylogenetic indices (Shannon index, Simpson index, and Inversed Simpson index) showed that the fall represented the highest microbial diversity while the artificial hibernation was the lowest (*t*-test, *p*-value < 0.05).

**Table 2:** Phylogenetic diversity indices of AH frogs and NH frogs

Diversity indices	NH frogs			AH frogs
	Fall	Winter	Spring	4 °C
Richness	375 ± 165^a^	350 ± 163^a^	282 ± 66^a^	91 ± 15^b^
Shannon index	3.50 ± 0.68^a^	3.45 ± 0.53^a^	3.46 ± 0.42^a^	1.88 ± 0.51^b^
Simpson index	0.90 ± 0.11	0.91 ± 0.05	0.91 ± 0.05	0.76 ± 0.11
Inverse Simpson index	16.58 ± 12.63^a^	14.86 ± 7.75^a^	15.26 ± 7.51^a^	4.80 ± 1.86^b^

*Abbreviations*: *AH frogs* artificially hibernating frogs, *NH frogs* nonhibernating frogs. Values are means ± SD. Within each row, values not sharing superscripts (a and b) differ significantly (*p*-value < 0.05, Student’s *t*-test)

### Compositional changes between AH and NH frogs

**Fig. 2:**
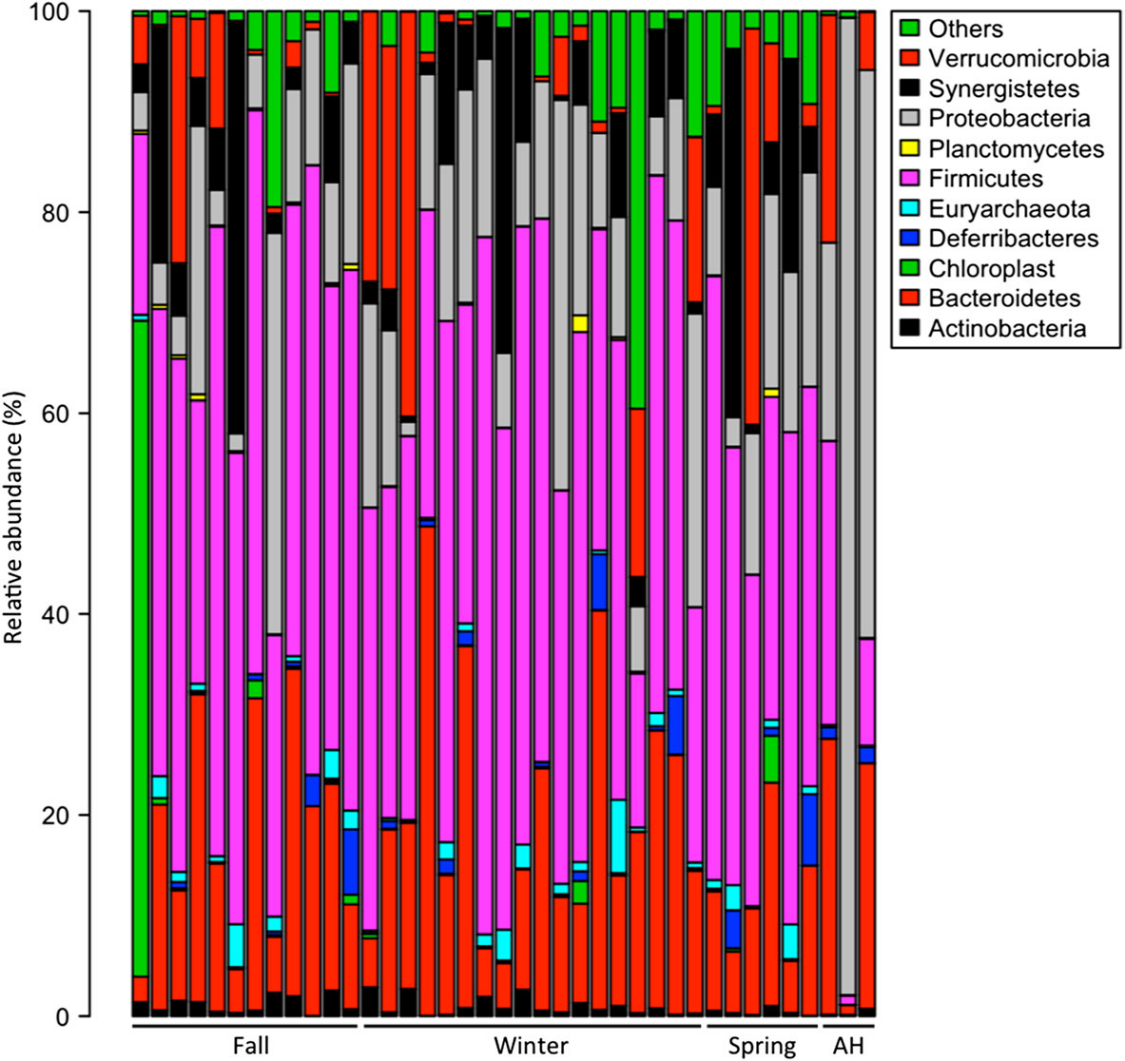
Taxonomic composition between AH and NH frogs. Bacterial taxonomic representation in fecal microbiota in AH frogs and NH frogs (fall, winter, and spring) at the phylum level

Microbial composition was similar among NH frogs, including fall, winter, and spring, except for the first individual in the fall showing that chloroplast dominated the fecal sample while others were dominated by Firmicutes (Fig. 2). There were no significant differences between fall, winter, and spring in relative abundance (on average) within NH frogs (*t*-test, *p*-value > 0.05). However, Firmicutes were significantly more abundant in NH frogs than in AH frogs (*t*-test, *p*-value < 0.05; Table 3). This suggests that artificial hibernation may alter some specific bacteria.

**Table 3:** Relative abundance of dominant phyla in AH frogs and NH frogs

OTUs	NH frogs			AH frogs
	Fall	Winter	Spring	4 °C
Bacteroidetes	17.07 ± 10.66	19.07 ± 12.45	11.82 ± 6.25	17.63 ± 14.49
Firmicutes	45.24 ± 13.82^a^	42.94 ± 13.56^a^	42.92 ± 10.56^a^	13.26 ± 13.84^b^
Fusobacteria	2.27 ± 5.22	4.67 ± 9.46	3.62 ± 3.76	0.23 ± 0.31
Proteobacteria	12.02 ± 11.59	14.98 ± 8.99	13.77 ± 6.87	57.82 ± 38.77
*Aeromonas*	0.09 ± 0.20	0.13 ± 0.24^a^	0.01 ± 0.02	0.001 ± 0.002^b^
*Citrobacter*	5.26 ± 8.58	3.11 ± 5.32^a^	3.55 ± 4.99	7.03 ± 0.57^b^
*Pseudomonas*	0.02 ± 0.07	0.01 ± 0.02	0.02 ± 0.02	13.48 ± 23.14
Verrucomicrobia	4.37 ± 7.23	7.65 ± 12.07	8.79 ± 15.45	9.50 ± 11.75

*Abbreviations*: *AH frogs* artificially hibernating frogs, *NH frogs* nonhibernating frogs. Values are means ± SD. Within each row, values not sharing superscripts (a and b) differ significantly (*p*-value < 0.05, Student’s *t*-test). The RLS-related genera listed in Methods differ in AH frogs compared with NH frogs are underlined

**Fig. 3:**
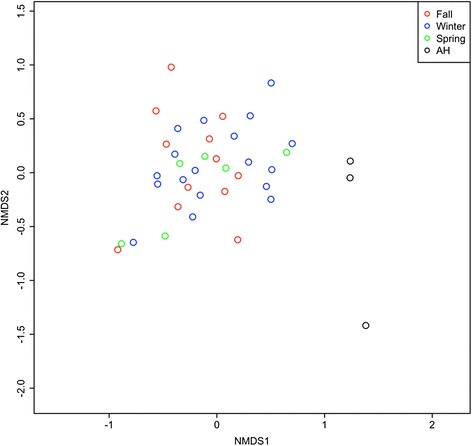
Compositional variation in microbial communities between AH and NH frogs. NMDS ordination between microbial communities of AH frogs and NH frogs (fall, winter, and spring). Each point represents an individual frog sample. *Red*, *blue*, *green*, and *black* colors refer to fall, winter, spring, and AH, respectively

**Fig. 4:**
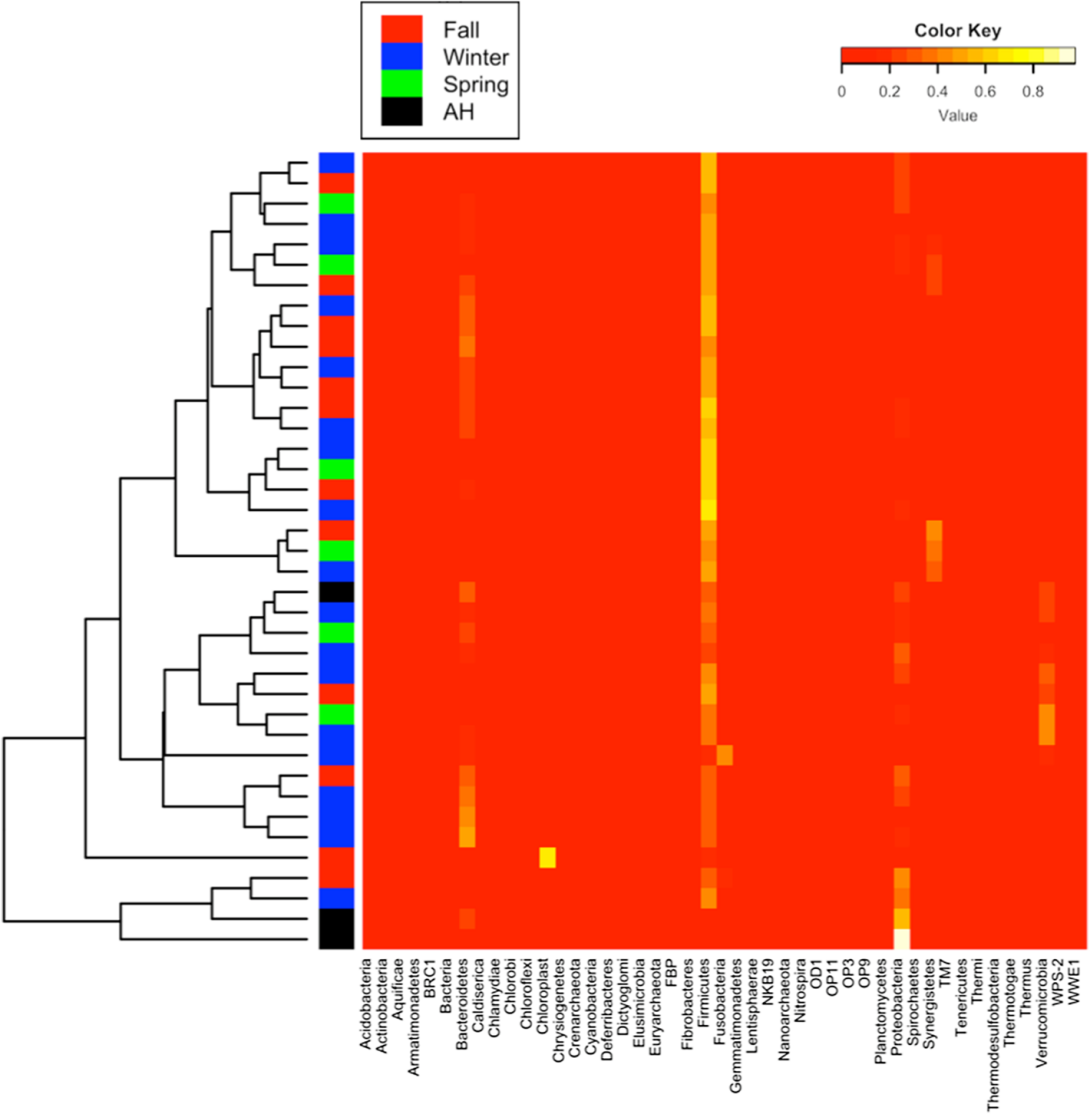
Heat map. A color-scale heat map demonstrates the relative abundance of bacterial phylotypes on the phylum level. *Red*, *blue*, *green*, and *black* colors on the left refer to fall, winter, spring, and AH, respectively

To reveal compositional change between AH and NH frogs, we conducted NMDS to compare microbial composition in the AH frogs with that in fall, winter, and spring frogs (Fig. 3). There is no difference in microbial composition between the fall, winter, and spring. However, we found that the AH frogs clustered separately with NH frogs (ANOSIM, *p* < 0.05). In addition, we compared the compositional similarity between AH and NH frogs by calculating the pairwise distance among OTU abundance (Fig. 4). Most of the NH frogs clustered together. This suggests that AH frogs contain distinct microbial composition compared with NH frogs.

### Core OTUs between AH and NH frogs

**Fig. 5:**
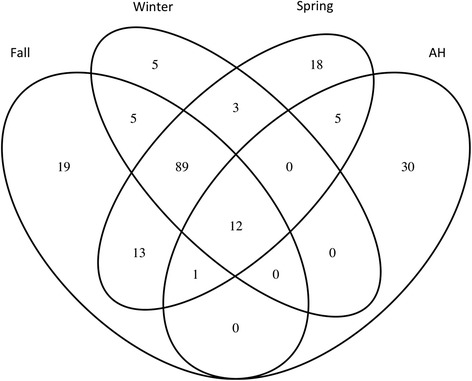
Core OTUs of AH frogs and NH frogs (fall, winter, and spring). Venn diagram summarizing the overlap of core OTUs of brown tree frog fecal microbiota in fall, winter, spring, and AH

To reveal whether AH frogs harbored a specific microbial composition to adapt to artificial hibernation, we compared core gut microbiota between AH and NH frogs. Core gut microbiota were defined as OTUs that were present on >80% of individual hosts in a population. Overall, a total of 139, 114, 141, and 48 core genera were observed in fall, winter, spring, and AH frogs, respectively (Fig. 5). Twelve core genera were shared among fall, winter, spring, and AH frogs. AH frogs harbored the highest ratio of core genera that were not present as core genera in NH frogs (63%, 30 of 48 core genera). This suggests that AH frogs may harbor certain microbes to regulate metabolic functions and facilitate adaptation of artificial hibernation.

### Changes in the amount of facultative, anaerobic bacteria, and RLS-related pathogens

Comparing eight facultative bacteria and ten anaerobic bacteria (listed in Methods) shared by AH and NH frogs, none of them showed significant differences in relative abundance. Considering the RLS-related pathogens of bullfrogs that were reported in previous studies, we found that the genus *Citrobacter* was significantly higher in relative abundance in AH frogs than NH frogs, while genus *Aeromonas* was significantly lower in AH frogs than NH frogs (*t*-test, *p*-value < 0.05; Table 3). Other RLS-related pathogens, such as *Staphylococcus* and *Streptococcus*, were not characterized in AH frogs and rarely distributed in NH frogs.

### AH frogs are exposed to a higher ratio of pathogens than NH by functional prediction via PICRUSt and Tax4Fun

We used PICRUSt to impute the metagenome from our 16S rRNA sequencing results. PICRUSt assignment of predicted metagenome content to Level 2 KEGG orthologs (KOs) suggested no significant functional differences within NH frogs (i.e., fall, winter, and spring). However, we found most predicted functional categories in the KEGG pathways are significantly change in AH frogs, compared with NH frogs (Level 2 KOs, Wilcoxon’s test, *p*-value < 0.05; Table 4), including amino acid metabolism and lipid metabolism. We also fount that KOs of infectious disease and immune system disease significantly increase in AH frogs, while environmental adaptation and signal transduction significantly decrease in AH frogs compared with NH frogs. In addition, KOs of *Vibrio cholera* infection, *Vibrio cholera* pathogenic cycle, pathogenic *Escherichia coli* infection, and primary immunodeficiency significantly increase in AH frogs compared with NH frogs via Tax4Fun (Wilcoxon’s test, *p*-value < 0.05; see Additional file 1). These results imply that artificial hibernation not only changes metabolism, environmental adaption, and environmental information processing, but also might be important to immune system and the activity of pathogenic invasion.

**Table 4:** PICRUSt showing predicted relative abundance of KEGG ortholog groups (Level 2 KOs)

KEGG pathways	NH frogs			AH frogs
	Fall	Winter	Spring	4 °C
Amino acid metabolism	9.169 ± 0.301^a^	9.222 ± 0.337^a^	9.084 ± 0.215^a^	10.855 ± 1.291^b^
Cell motility	4.297 ± 0.552^a^	4.338 ± 0.508^a^	4.674 ± 0.238^a^	3.258 ± 0.3^b^
Cellular processes and signaling	3.572 ± 0.107	3.64 ± 0.135	3.702 ± 0.123^a^	3.488 ± 0.102^b^
Circulatory system	0.005 ± 0.004	0.004 ± 0.003^a^	0.004 ± 0.005	0.025 ± 0.026^b^
Endocrine system	0.265 ± 0.018^a^	0.273 ± 0.026^a^	0.258 ± 0.02^a^	0.463 ± 0.196^b^
Environmental adaptation	0.165 ± 0.013^a^	0.169 ± 0.013^a^	0.172 ± 0.012^a^	0.149 ± 0.004^b^
Enzyme families	2.224 ± 0.059^a^	2.216 ± 0.052	2.155 ± 0.128	1.946 ± 0.391^b^
Folding, sorting and degradation	2.092 ± 0.107^a^	2.162 ± 0.173^a^	2.095 ± 0.142^a^	2.384 ± 0.065^b^
Genetic information processing	2.404 ± 0.081	2.472 ± 0.118	2.507 ± 0.121^a^	2.321 ± 0.072^b^
Immune system diseases	0.03 ± 0.006^a,c^	0.029 ± 0.006^a,c^	0.024 ± 0.005^b^	0.036 ± 0.007^c^
Infectious diseases	0.312 ± 0.013^a^	0.318 ± 0.016^a^	0.319 ± 0.031	0.394 ± 0.055^b^
Lipid metabolism	2.759 ± 0.107^a^	2.733 ± 0.128^a^	2.71 ± 0.112^a^	3.96 ± 1.373^b^
Membrane transport	14.685 ± 1.045^a^	14.072 ± 1.519^a^	14.645 ± 1.177^a^	9.828 ± 2.509^b^
Metabolism	2.492 ± 0.057^a^	2.505 ± 0.068^a^	2.492 ± 0.065^a^	2.616 ± 0.102^b^
Metabolism of other amino acids	1.479 ± 0.08^a^	1.463 ± 0.077^a^	1.442 ± 0.107^a^	2.042 ± 0.516^b^
Metabolism of terpenoids and polyketides	1.353 ± 0.115^a^	1.384 ± 0.142^a^	1.351 ± 0.134^a^	2.282 ± 0.884^b^
Nervous system	0.093 ± 0.003^a^	0.092 ± 0.003^a^	0.091 ± 0.003^a^	0.104 ± 0.008^b^
Poorly characterized	4.872 ± 0.083^a^	4.909 ± 0.15	4.912 ± 0.135	5.216 ± 0.403^b^
Signal transduction	2.15 ± 0.171^a^	2.194 ± 0.18^a,b,d^	2.302 ± 0.126^b^	1.926 ± 0.111^c^
Transcription	2.875 ± 0.159^a^	2.765 ± 0.248^a^	2.834 ± 0.262^a^	2.193 ± 0.342^b^
Xenobiotics biodegradation and metabolism	1.75 ± 0.152^a^	1.709 ± 0.177^a^	1.789 ± 0.306^a^	3.463 ± 2.176^b^

*Abbreviations*: *AH frogs* artificially hibernating frogs, *NH frogs*, nonhibernating frogs. Values are means ± SD. Within each row, values not sharing superscripts (a, b, c, and d) differ significantly (*p*-value < 0.05, Wilcoxon’s test)

## Discussion

Some intestinal microbiota and their host develop a strong relationship. Studies have shown that artificial hibernation alters gut microbiota and is able to cause pathogen-induced disease, such as septicemia, due to the rise of pathogenic bacteria triggered by chilling to close to 4 °C [17, 19]. The effects on gut microbiota of the slower metabolism and nutrient turnover that are triggered by hibernation still lack a comprehensive analysis in amphibians. To explain compositional and functional shifts that were triggered by hibernation, we revealed the change of gut microbiota between AH and NH frogs by metagenomic analysis. We calculated several diversity indices, NMDS, heat map to infer alpha, beta diversity, and the change of gut microbiota between AH and NH frogs. The number of species and evenness within the community are usually thought to affect the biodiversity–ecosystem functioning relationship, as well as functional traits and their interaction [44–48]. Therefore, we further investigated the changes in microbial composition and predicted their function by PICRUSt and Tax4Fun.

Ecosystem functioning is often positively correlated with microbial composition and species richness [49–53]. Our results demonstrated that all diversity indices in AH frogs were significantly smaller than in NH frogs, suggesting that artificial hibernation in frogs may reduce microbial ecosystem functioning in the gastrointestinal tract accompanied by slower metabolism. There were no significant changes in microbial composition within NH frogs (i.e., fall, winter, and spring). However, both NMDS and heat map showed that microbial compositions in AH frogs were significantly different from those in NH frogs, reflecting that artificial hibernation could alter gut microbiota. This reconstruction of microbial community in the frog gut might potentially modify species interactions for ecosystem functioning.

It is also worth noting that the distribution of dominant bacteria found in frogs is different from that found in fish. The most dominant phyla in the brown tree frog were Bacteroidetes, Firmicutes, and Proteobacteria, whereas the relative abundance of Bacteroidetes, Firmicutes, and Proteobacteria in the leopard frog represented 22.8 ± 8.96, 66.05 ± 8.90, and 10.43 ± 3.39%, respectively [30], suggesting that the major bacterial phyla were consistent between brown tree frog and leopard frog. On the other hand, several studies showed that the relative abundance of Bacteroidetes is rarely represented in carp and Nile tilapia [54–56]. Another comprehensive analysis that collected 25 fish species in the GenBank library also showed consistent results that fish harbored Proteobacteria (63%) in high relative abundance, whereas Bacteroidetes remained relatively low on average (6%) [57]. Therefore, our data indicated that amphibians might host different dominant microbes compared with that in fish.

We further compared the unique and shared OTUs of gut microbiota between frogs and a mammal hibernator, the ground squirrel. We first compared the AH brown tree frogs with leopard frogs, which characterized microbial composition by culture counts and isolation [16]. We found a consistent result in these two frogs, for example, genus *Pseudomonas* formed a larger proportion of gut microbiota in AH frogs than NH frogs both in brown tree frogs and leopard frogs. However, in the case of ground squirrels, *Pseudomonas* showed no significant difference in relative abundance between hibernating and nonhibernating states. In addition, the phyla Bacteroidetes and Verrucomicrobia significantly increased in their relative abundance in hibernating ground squirrels compared with nonhibernating ground squirrels, where these two phyla showed no significant differences between AH and NH frogs [8]. Our results suggest that gut microbiota tend to show a unique response to hibernation between amphibian and mammal.

According to our function analysis using 16S rRNA profile, we found that artificial hibernation may cause frog samples to be exposed to potential pathogens, leading to disease. For example, RLS, an infectious disease caused by septicemia, was the main cause of frog mortality [38–40]. Pathogens that increase the risk of RLS included *Pseudomonas aeruginosa* and *Staphylococcus epidermidis* [39, 40, 58]. Although, the RLS-related pathogens in brown tree frogs are still uncharacterized. Here, we found that the relative abundance of a few RLS-related pathogenic genera that were reported in bullfrogs [43, 59, 60] represented higher in AH frogs than NH frogs. For example, genus *Citrobacter* showed significantly higher in AH frogs than NH frogs. The relative abundance of genus *Pseudomonas* represented higher in AH frogs than in NH frogs. Other RLS-related pathogens described in bullfrogs, such as *Staphylococcus* and *Streptococcus*, rarely contributed in brown tree frogs. We also found that microbes in AH frogs were dominated with the pathways corresponding to the genes for bacterial invasion. These results imply that the pathways corresponding to the genes for bacterial invasion might show higher adaptation in artificial hibernation. Therefore, the result might explain that some of the pathogenic populations increase in their relative abundance. This might lead to a higher mortality rate during chilling and hibernation reported in previous studies [19, 20] and cause the pathogenic invasion.

An interesting case is *Laribacter hongkongensis*, a facultative anaerobic bacterium, was found to be associated with community-acquired gastroenteritis [61–63]. *L. hongkongensis* was a discovered bacterial genus and species first isolated from the blood and empyema pus of a man with alcoholic cirrhosis and bacteremic empyema thoracis in Hong Kong [64]. In the previous studies, *L. hongkongensis* was found to be highly distributed in natural freshwater environments and freshwater fish, such as grass carp (60% recovery rate), bighead carp (53% recovery rate), mud carp, and large-mouth bass [65, 66]. In 2009, a population of *L. hongkongensis* was also highly isolated (80% recovery rate) in amphibians by pulsed-field gel electrophoresis [67]. This evidence implies that the bacterium is well adapted to different freshwater environments and freshwater animals. However, *L. hongkongensis* was present but rare in brown tree frogs, suggesting that the brown tree frog might be able to resist this pathogen.

Hibernation is associated with a dramatic remodeling of many intestinal functions, such as energy and intestinal immune system, in both small and large hibernators. For example, an enrichment of Bacteroidetes and lower relative abundance of Firmicutes has previously been observed in the microbiota of hibernators [7–9, 68, 69]. The increase in Bacteroidetes may be explained by their capacity to switch their metabolism toward degradation of host glycans in the absence of dietary polysaccharides [70] or their capacity to metabolize protein and fat [71] putatively provided by the intestinal epithelium. In our results, although Bacteroidetes did not significantly increase in AH frogs compared with NH frogs, KEGG pathways relative metabolism increase in AH frogs. This suggests that microbiota may contribute to host energy metabolism in the hibernating brown tree frog, and these results can only be observed by functional analysis.

In addition, recent studies showed that seasonal reorganization of the microbiota is a major driver of the immune alterations because the immune system is the primary sensor of gut microbes and their metabolites [72, 73]. Mucins are a family of polydisperse molecules designed to carry out multiple tasks at the mucosal surface of the gastrointestinal tract. The mucosal surface throughout the gastrointestinal tract must resist the aggressive elements from the external environment present in the diet. The mucus defensive barrier forms the first line of defense to the external environment and contains both innate and adaptive immune elements [74, 75]. Therefore, the defective mucus barrier with increased permeability result in inflammation [74, 76] and may increase the risk of pathological infection. Our results show that AH frogs did not significantly increase the relative abundance of the mucin-degrader, *Akkermansia*. However, the functional predictions via PICRUSt and Tax4Fun showed that AH frogs not only significantly increase KEGG pathways in infectious disease, but also significantly decrease signal transduction. Immune changes are often associated with altered host–microbe signaling [72, 77–79], and there is significant evidence showing that certain cytokines are involved in not only the initiation but also the persistence of pathogenic pain [80]. Therefore, by applying functional analysis using 16S rRNA profiles, we suggest that hibernation may impose potential effects on primary immunodeficiency and increase the risk of bacterial infections in the brown tree frog.

## Conclusions

Artificial hibernation in the brown tree frog reduced microbial diversity and levels of Firmicutes in the intestinal tracts. AH frogs tend to harbor core OTUs that are rarely distributed among NH frogs. Artificial hibernation also increased the relative abundance of RLS-related pathogens, such as *Citrobacter* and *Pseudomonas*. Functional predictions via PICRUSt and Tax4Fun infer that AH frogs change pathways corresponding to the genes for metabolism, organismal system, information processing, and disease. We infer that artificial hibernation may impose potential effects on bacterial invasion and primary immunodeficiency.

## Additional file

Additional file 1: Tax4Fun showing predicted relative abundance of KEGG ortholog groups. Abbreviations: AH frogs, artificially hibernating frogs; NH frogs, nonhibernating frogs. Values are means ± SD. (XLSX 31 kb)

## Abbreviations

AH frogs: Artificially hibernating frogs
NH frogs: Nonhibernating frogs
OTU: Operational taxonomic unit
